# Proximal Femoral Morphology in Development Dysplasia of the Hip Based on Three-Dimensional (3D) Analysis

**DOI:** 10.5704/MOJ.2507.014

**Published:** 2025-07

**Authors:** T Tachibana, H Katagiri, T Ogawa, K Miyatake, R Takada, T Jinno

**Affiliations:** 1 Department of Orthopaedic Surgery, Dokkyo Medical University Saitama Medical Center, Koshigaya, Japan; 2 Department of Orthopaedic Surgery, Tokyo Medical and Dental University Hospital, Tokyo, Japan; 3 Department of Health Policy and Informatics, Tokyo Medical and Dental University Hospital, Tokyo, Japan; 4 Department of Joint Surgery and Sports Medicine, Tokyo Medical and Dental University Hospital, Tokyo, Japan

**Keywords:** arthroscopy, femoroacetabular impingement, hip dysplasia, osteotomy, proximal femoral morphology

## Abstract

**Introduction:**

Surgeons performing periacetabular osteotomy (PAO) should account for proximal femoral morphology to prevent secondary femoroacetabular impingement. Herein, we aimed to clarify proximal femoral morphology in patients with developmental dysplasia of the hip (DDH).

**Materials and methods:**

This retrospective study included 57 patients with DDH (77 hips) who underwent PAO (DDH group). The control group comprised 30 patients (30 hips) with unilateral femoral head necrosis and contralateral unaffected hips (healthy hips). Coronal planes were created parallel to the femoral neck axis based on three-dimensional image analysis of hip computed tomography images. Coronal slices were obtained using clockwise rotation around the femoral neck axis in 15° increments, creating seven positions for measuring alpha (α)-angles. The superior and anterior directions were defined as 12 o'clock and 3 o'clock, respectively. Cam deformity was defined as an α-angle ≥60°. Outcome measurements were the α-angles of seven slices, cam deformity, and correlations between the maximum value of the α-angles and related factors.

**Results:**

α-Angles were greater in the superior direction in the control than in the DDH group; conversely, they were greater in the anterior direction in the DDH than in the control group. The DDH group had more cam deformities than the control group. Cam deformities were more superior (12:30 to 1:00) in the control group, and more anterior (2:00 to 3:00) in the DDH group. Maximum α-angles in the DDH group correlated with superior acetabular coverage.

**Conclusion:**

Surgeons should carefully consider acetabular version during PAO and avoid acetabular retroversion in cases with cam deformities.

## Introduction

Periacetabular osteotomy (PAO) is the standard treatment for correcting acetabular coverage and preventing osteoarthritis in patients with developmental dysplasia of the hip (DDH)^1^. Although several studies have reported good long-term results after PAO, this procedure does not completely prevent the progression of osteoarthritis or poor outcomes^2,3^. A previous study emphasised the importance of selecting patients who can fully benefit from PAO to prevent failure^4^. Demographic and radiographic risk factors for the progression of osteoarthritis after PAO include advanced age at surgery, severe hip dysplasia, pre-operative osteoarthritis, and poor joint congruity^3,5^.

Myers *et al* reported that acetabular overcorrection or acetabular retroversion after PAO could cause secondary femoroacetabular impingement (FAI)^6^. Moreover, Albers *et al* reported that secondary FAI affects 10-year survival after PAO^7^. Therefore, for proper acetabular coverage correction, PAO should be performed based on the three-dimensional (3D) morphology of the acetabulum and proximal femur^8^. Previous analyses based on computed tomography (CT) data reported the morphological details of acetabular deficiency in dysplastic hips^9,10^, particularly, the femoral anteversion^11^; however, less is known about the proximal femoral morphology in DDH.

Therefore, this study aimed to clarify the proximal femoral morphology and the prevalence of cam deformities in patients with DDH. We hypothesised that cam deformities would occur in a more anterior direction in patients with DDH.

## Materials And Methods

This retrospective study was approved by our institutional review board and performed in accordance with the Helsinki Declaration of 1975 (as revised in 1983). Sixty-four consecutive patients with DDH (84 hips) who underwent PAO of one or both hips to treat hip dysplasia between January 2001 and April 2018 were initially enrolled in this study. The surgical indications for PAO were as follows: (1) persistent hip pain after conservative treatment for at least 3 months, (2) lateral centre-edge Wiberg angle of <25º, and (3) a closed epiphyseal line. The contraindications for PAO were (1) advanced osteoarthritis above Tönnis grade 2 and (2) no improvement in joint congruity or femoral head coverage by maximum hip abduction on radiography^12^. The exclusion criteria were as follows: (1) prior pelvic surgery and (2) absence of pre-operative CT scans. We excluded seven patients (seven hips). Of these, one patient had undergone prior surgery, and six patients (six hips) did not undergo preoperative CT scans. The remaining 57 patients (77 hips) were assigned to the DDH group (Table I).

**Table I:** Patient characteristics in the DDH and control groups.

	DDH (n=77 hips)	Control (n=30 hips)	p-value
Age, years*	33.2 ± 11.0 (range 13-55)	35.9 ± 13.1 (range 15-59)	0.37
Sex, female, (%)†	72 (94)	29 (97)	0.52
Body mass index, (kg/m^2^)*	22.5 ± 3.5 (range 15.1-32)	22.8 ± 3.3 (range 18-29)	0.87
Centre-Edge angle, (°)*	9.2 ± 7.8 (range -8 to 24)	32.6 ± 4.5 (range 26-41)	<0.001

Note: ^*^Mann-Whitney U test : †Pearson’s chi-square test : DDH, developmental dysplasia of the hip

We reviewed the pre-operative anterior/posterior (AP) radiographs, magnetic resonance images (MRI), and CT scans of the pelvis in 129 patients (129 hips) with unilateral steroid-associated femoral head necrosis who were examined between January 2010 and April 2018. The contralateral hip was without pathology, i.e., non-dysplastic. These individuals served as the control group. The reason for selecting patients with steroid-associated femoral head necrosis as the control group was that their male-to-female ratio and their age at diagnosis were comparable to those of the DDH group^13^. The inclusion criteria for this group were patients younger than 60 years of age whose unaffected side of the hip showed no femoral head necrosis or Stage I necrosis according to the Association Research Circulation Osseous staging system^14^. Thirty-three patients (33 hips) met these criteria. The exclusion criterion was hip dysplasia, which was defined as a lateral centre-edge angle of Wiberg <25° on AP pelvic radiographs. Three patients (three hips) with dysplasia were excluded. Based on these criteria, 30 patients (30 hips) were included in the control group (healthy hip group). The demographic characteristics of both groups are shown in Table I. Medical records were retrospectively reviewed to collect demographic data including age, sex, and body mass index (BMI) at the time of examination. Preoperative hip range of motion (ROM) for flexion and abduction was reviewed in the DDH group.

**Fig. 1:**
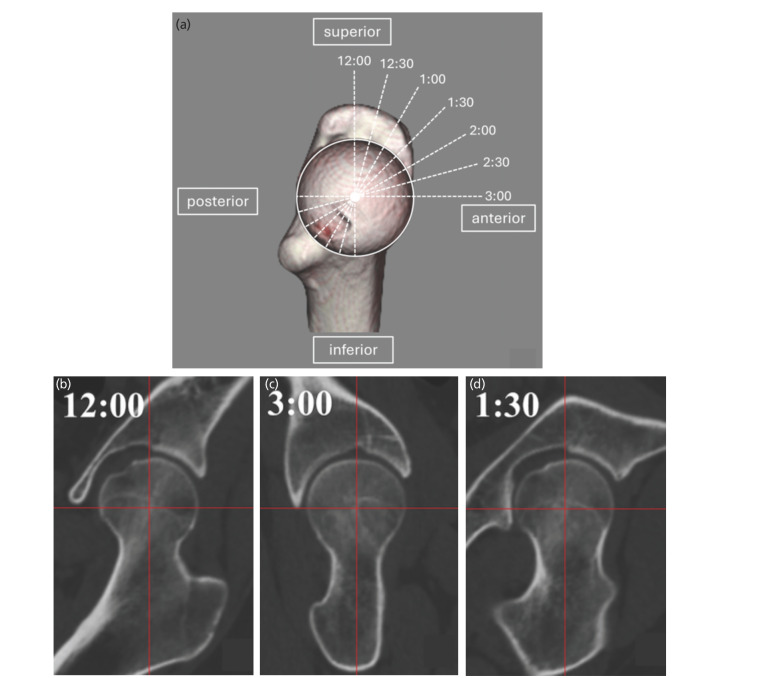
Computed tomography images of the left hip. (a) Seven increments perpendicular to the femoral neck axis were chosen for measurements of the alpha angles in each radial direction. (b) Coronal slices at the 12:00 clock position, (c) the 1:30 position, and (d) the 3:00 clock position.

**Fig. 2:**
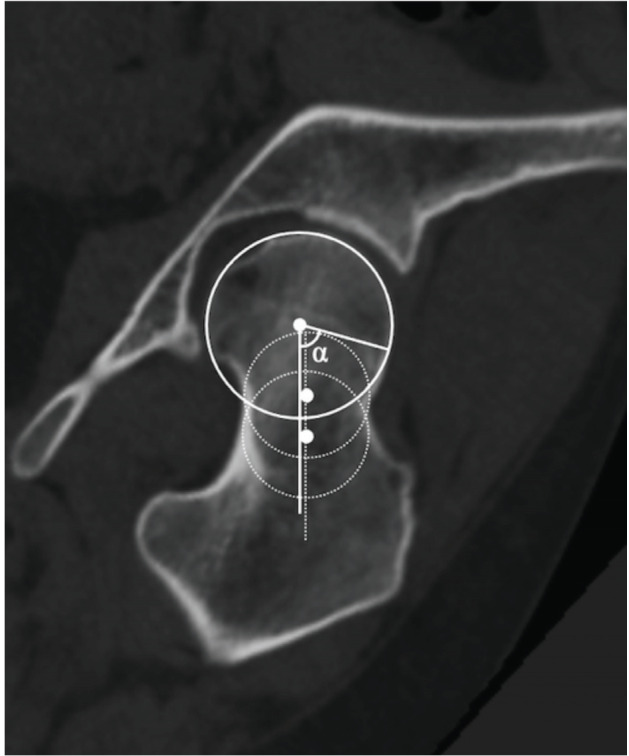
Measurement of the alpha angle in the radial direction in the 1 o’clock position. The axis of the femoral neck was defined as the line passing through the centre of the femoral head, running parallel to the dotted line connecting the centres of the two circles positioned over the femoral neck.

Pre-operative CT scans were obtained at the level of the iliac wing to the femoral condyle. The slice thickness was approximately 1mm, and the CT data were transferred to ZedHip [Lexi, Tokyo, Japan], 3D templating software package. We created a coronal plane running parallel to the femoral neck axis, including the centre of the femoral head, based on 3D image analysis of hip CT images. Each coronal slice was obtained using clockwise anterior rotation in 15° increments around the femoral neck axis. These slices provided seven clockface positions for measuring the α-angles (Fig. 1). The superior direction was defined as 12 o'clock and the anterior direction as 3 o'clock. The method proposed by Bouma *et al*^15^ was used for measurements of the α-angles at each clockface position (Fig. 2). A cam deformity was defined as an α-angle ≥60°^16^. Cam deformity was diagnosed when the α-angle was ≥60° in any one of the seven positions. The maximum value of the α-angle among the seven positions was recorded.

**Fig. 3:**
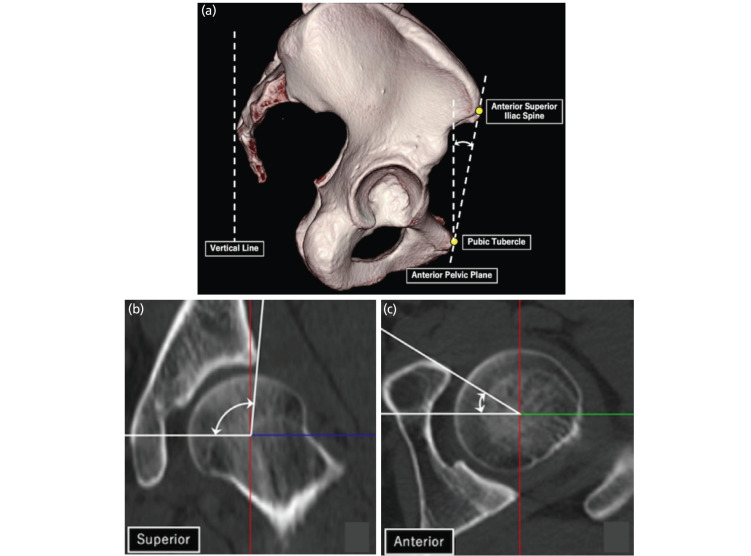
Measurement of acetabular parameters in the functional pelvic position. (a) The anterior pelvic plane angle was defined as the angle between the vertical line and the anterior pelvic plane. (b, c) Superior and anterior acetabular coverage was defined as the angle between a line connecting the acetabular edge and the centre of the femoral head, and a horizontal line.

To investigate correlations with the α-angle, acetabular parameters and femoral anteversion were measured using CT data as follows. The pelvic position was standardised with reference to the functional pelvic plane coordinate system^17^. The anterior pelvic plane (APP) angle in the supine position was measured as an indicator of pelvic sagittal tilt and defined as the angle between the horizontal plane and the APP, including the bilateral anterosuperior iliac spines and the most anterior point of the pubic symphysis (Fig. 3)^18^. Acetabular coverage was defined as the angle between the line connecting the acetabular edge and the centre of the femoral head and a horizontal line^10^. The superior and anterior acetabular coverage was measured (Fig. 3). The anterior centre-edge angle and acetabular anteversion were quantified in sagittal and transverse sections through the centre of the femoral head, respectively. The acetabular version was quantified on the axial plane 5mm distal to the acetabular roof^19^. Femoral anteversion was measured in the retrocondylar plane^20^.

Statistical analyses were performed using STATA 16 software [StataCorp LP, College Station, TX, USA]. All data are presented as means ± standard deviations (SD). The Mann–Whitney U test was used to compare continuous parameters between the DDH and control groups. Chi-square analyses were used to assess the associations between discrete variables. Correlations of the maximum value of the α-angles with continuous parameters (e.g., age, BMI, APP angle, acetabular coverage, anterior centre-edge angle, acetabular version, and femoral anteversion) in patients with DDH were calculated using Spearman’s rank correlation coefficients. The level of significance was set at p<0.05.

## Results

The demographic characteristics of the DDH and control groups are shown in Table I. The patients in the DDH group had smaller centre-edge angles than the control group. No significant differences were found between the groups in age, sex, or BMI. The overall pre-operative ROM in the DDH group was 112 ± 10° for flexion and 29 ± 4° for abduction.

**Fig. 4:**
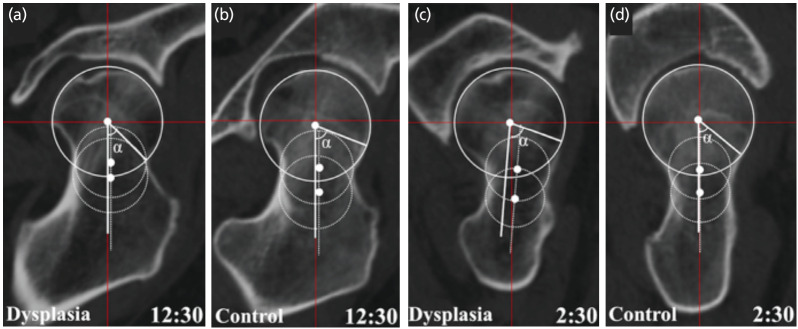
(a) Measurement of the alpha angle in the dysplasia group and (b) control group at the 12:30 clock position, and (c) in the dysplasia group and (d) control group at the 2:30 clock position. The alpha angle was greater in the control group at the 12:30 clock position, whereas it was greater in the dysplasia group at the 2:30 clock position.

Table II shows the α-angles in each direction in both groups. The control group showed a significantly greater α-angle in the superior direction (at 12:30) than that in the DDH group (p=0.019), as shown in Fig. 4a and 4b. Conversely, the DDH group showed a significantly greater α-angle in the anterior direction (at 2:30 and 3:00) than that in the control group (p=0.0021, 0.025, respectively), as shown in Fig. 4c and 4d.

**Table II:** The α-angle at each direction in the DDH and control groups.

Direction	DDH	Control	p-value
12:00 (Superior), (°)*	40.9 ± 4.0	41.8 ± 3.1	0.097
12:30, (°)*	43.9 ± 4.0	47.1 ± 6.2	**0.019**
1:00, (°)*	47.5 ± 4.8	49.8 ± 8.1	0.20
1:30, (°)*	50.9 ± 6.7	50.8 ± 7.6	0.92
2:00, (°)*	53.3 ± 7.9	50.0 ± 6.6	0.051
2:30, (°)*	52.8 ± 10.5	46.1 ± 6.6	**0.0021**
3:00 (anterior), (°)*	47.7 ± 11.6	41.7 ± 5.8	**0.025**
Maximum value of α-angles	57.5 ± 9.2	53.8 ± 6.9	0.051

Note: * Mann-Whitney U test : Statistically significant p-values (p<0.05) are shown in bold : DDH, developmental dysplasia of the hip; α, alpha

**Fig. 5:**
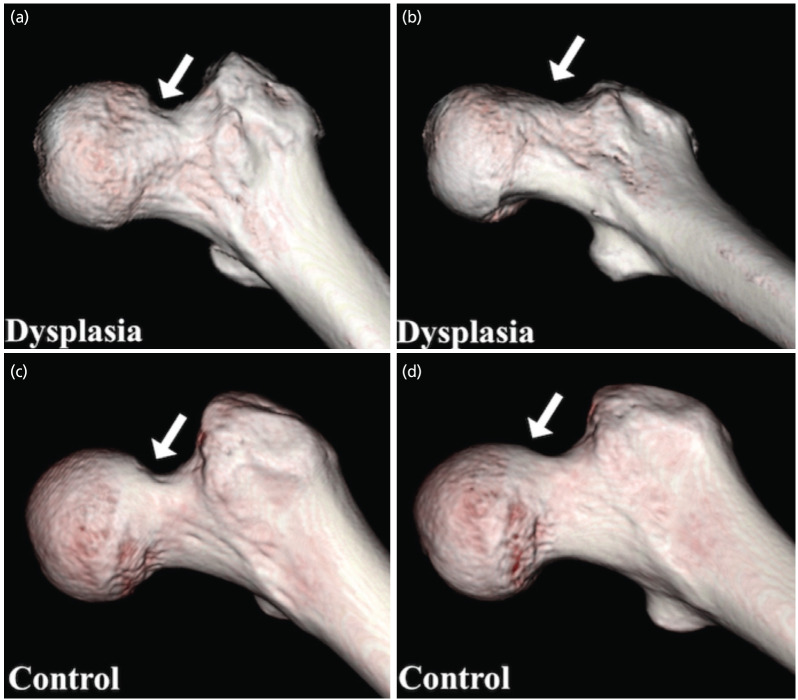
(a) Cam deformities in the DDH group (indicated by arrows) at 40° of abduction and (b) at 40° of abduction plus 20° of flexion. (c) Cam deformities in the control group (indicated by arrows) at 40° of abduction and (d) at 40° of abduction plus 20° of flexion.

Table III shows the prevalence of cam deformities in each direction in both groups. The DDH group had significantly more cam deformities than the control group in all directions (35.1% vs. 13.3%, p=0.026). The cam deformities in the DDH group occurred more anteriorly (from 2:00 to 3:00) than those in the control group, as shown in Fig. 5a and 5b. In contrast, the cam deformities in the control group occurred more superiorly (at 12:30 and 1:00) than those in the DDH group, as shown in Fig. 5c and 5d.

**Table III:** Cam deformity prevalence at each direction in the DDH and control groups.

Direction	DDH	Control	p-value
12:00 (Superior), (%)*	0	0	1.0
12:30, (%)*	0	6.7	0.022
1:00, (%)*	1.3	10.0	0.033
1:30, (%)*	6.5	6.7	0.97
2:00, (%)*	24.7	3.3	0.011
2:30, (%)*	24.7	6.7	0.035
3:00 (anterior), (%)*	16.9	0	0.016
All direction, (%)*	35.1	13.3	0.026

Note: * Pearson’s chi-square test : Statistically significant p-values (p<0.05) are shown in bold. : DDH, developmental dysplasia of the hip

Table IV shows the other radiographic parameters of the DDH and control groups. Patients in the DDH group demonstrated significantly smaller superior and anterior acetabular coverage and anterior centre-edge angles than those in the control group. Furthermore, patients in the DDH group showed a significantly greater acetabular version and femoral anteversion than those in the control group. There was no significant difference in the APP angle between the two groups.

**Table IV:** Radiographic measurements in the DDH and control groups.

Parameter	DDH	Control	p-value
Superior acetabular coverage, (°)*	102.3 ± 7.8	123.7 ± 5.6	<0.001
Anterior acetabular coverage, (°)*	44.7 ± 7.8	58.6 ± 8.8	<0.001
Acetabular anteversion, (°)*	13.3 ± 8.1	5.3 ± 10.7	<0.001
Anterior centre-edge angle, (°)*	41.6 ± 14.5	61.3 ± 7.0	<0.001
Anterior pelvic plane angle, (°)*	6.5 ± 4.9	6.0 ± 5.7	0.73
Femoral anteversion, (°)*	29.5 ± 13.5	19.0 ± 12.0	<0.001

Note: * Mann-Whitney test : DDH, developmental dysplasia of the hip

Table V shows the correlation between the maximum value of the α-angles and demographic and CT measurement parameters. In the DDH group, the maximum value of the α-angles significantly correlated with superior acetabular coverage (r = -0.29; p=0.018). No significant correlations were observed with the other factors. The maximum value of the α-angles did not show significant correlations with any factors in the control group. No significant differences were observed in the ROM between the patients in the DDH group with and those without cam deformities (flexion, 112° vs 113°, p=0.86; abduction 29° vs 30°, p=1.0, respectively). Furthermore, the correlation analysis between the maximum α-angle and pre-operative ROM in the DDH group revealed no significant correlations. The correlation coefficient for flexion was -0.01 (p=0.94), and the correlation coefficient for abduction was 0.11 (p=0.44).

**Table V:** Correlation between the maximum value of α-angles and demographic and computed tomography measurement parameters.

Parameter	The maximum value of α-angles in DDH group	The maximum value of α-angles in control group
Age	-0.04 (0.74)	0.20 (0.43)
Body mass index	0.08 (0.54)	0.1 (0.69)
Superior acetabular coverage	-0.29 (0.018)	0.35 (0.17)
Anterior acetabular coverage	-0.12 (0.35)	0.02 (0.94)
Acetabular version	0.01 (0.96)	0.17 (0.50)
Anterior centre-edge angle	-0.03 (0.79)	-0.20 (0.45)
Anterior pelvic plane angle	0.17 (0.19)	-0.12 (0.65)
Femoral anteversion	-0.12 (0.34)	-0.05 (0.83)

Note: Values are presented as correlation coefficients (p-value). : Statistically significant p-value (p < 0.05) is shown in bold. : DDH, developmental dysplasia of the hip; α, alpha

## DISCUSSION

The most important finding in this study was that 35% of the hips of the patients with DDH who required PAO had cam deformities, and the direction of the deformity in the patients with DDH was more anterior than that of patients in the control group.

Paliobeis *et al* reported that 47% of patients with FAI also had some elements of DDH radiographically, and their findings, based on plain radiographs, are similar to those of the present study^21^. However, their study did not show a prevalence of cam deformities in patients with acetabular dysplasia. Ida *et al* reported that 40% of the patients with DDH who underwent PAO had cam deformities^22^. Although their results were based on plain radiographs, they were also consistent with the results of the present study. Using CT data in our study enabled us to reveal the morphology of the proximal femur in patients with DDH via 3D analysis. Our results showed that approximately 1/3 of patients with DDH requiring PAO had cam deformities in the anterior direction. These results suggest that surgeons should specifically avoid retroversion of the acetabulum when performing PAO.

Kobayashi *et al* reviewed the MRIs of 20 hip joints in patients with borderline DDH and reported that the direction of the maximum α-angle of the hips of patients with borderline DDH was more anterior than that of patients with FAI^23^. Although their report was based on a small number of cases, collectively, their results combined with ours indicate that the shape of the proximal femur may be similar in patients with borderline DDH treated with hip arthroscopy and in those treated with PAO. Hip arthroscopy can be successful when appropriately performed with a precise target. However, in patients with hip dysplasia, arthroscopy could cause devastating consequences^24,25^. Capsulotomy during hip arthroscopy is critical for obtaining an excellent view and avoiding incomplete treatment of cam deformities; however, this procedure can cause iatrogenic instability, particularly in patients with DDH^26-28^. Our detailed information about cam deformities in patients with DDH may encourage arthroscopic surgeons to more carefully consider the ideal placement and size of the capsulotomy, thereby reducing the risk of iatrogenic instability.

The results of the present study suggest that the maximum value of the α-angle may correlate with smaller superior acetabular coverage in patients with DDH. A cam deformity in patients with FAI is thought to be increased by repeated collisions between the acetabular rim and femoral neck^29^. Whether impingement occurs anteriorly in the proximal femur in patients with DDH due to significantly more shallow superior acetabular coverage or whether the mechanism of the cam deformity development might be different between patients with FAI and those with DDH remains unclear. To clarify these issues, long-term cohort studies with large sample sizes are needed.

A strength of this study was the accuracy of the measurements, which allowed us to detail the magnitude and direction of the cam deformities in the DDH and control groups. However, this study had several limitations. First, this was a single-centre retrospective study; therefore, our findings are based on a relatively small sample size. Multicentre studies with large sample sizes are needed to fully elucidate the proximal femoral morphology in patients with DDH. Second, the control group which represented the “healthy hip” group, had hips with osteonecrosis of the femoral head. Normally, the hip joints of healthy volunteers without contralateral hip pathology are used as controls. Third, we did not assess the positive rate of the anterior impingement test in this study. However, Wells *et al* reported no significant difference in impingement test outcomes between DDH patients with and without cam deformities^8^. This indicates that diagnosing cam deformities using clinical tests alone may be challenging in patients with DDH.

Our study revealed a 35% prevalence of cam deformities in patients with DDH. Cam deformities were oriented more anteriorly in patients with DDH. These findings suggest that surgeons should carefully consider acetabular version during PAO and avoid acetabular retroversion in cases with cam deformities.

## Conclusion

Surgeons are reminded to consider assessing the acetabular version during the Peri-Acetabular Osteotomy (PAO) and avoid acetabular retroversion in cases with the cam deformities. This is to prevent secondary femoro-acetabular impingment.
